# Echoic memory of a single pure tone indexed by change-related brain activity

**DOI:** 10.1186/1471-2202-11-135

**Published:** 2010-10-20

**Authors:** Koji Inui, Tomokazu Urakawa, Koya Yamashiro, Naofumi Otsuru, Yasuyuki Takeshima, Makoto Nishihara, Eishi Motomura, Tetsuo Kida, Ryusuke Kakigi

**Affiliations:** 1 Department of Integrative Physiology, National Institute for Physiological Sciences, Okazaki 444-8585, Japan; 2 Multidisciplinary Pain Center, Aichi Medical University, Aichi 480-1195, Japan; 3 Department of Psychiatry, Mie University Graduate School of Medicine, Tsu Mie 514-8507, Japan

## Abstract

**Background:**

The rapid detection of sensory change is important to survival. The process should relate closely to memory since it requires that the brain separate a new stimulus from an ongoing background or past event. Given that sensory memory monitors current sensory status and works to pick-up changes in real-time, any change detected by this system should evoke a change-related cortical response. To test this hypothesis, we examined whether the single presentation of a sound is enough to elicit a change-related cortical response, and therefore, shape a memory trace enough to separate a subsequent stimulus.

**Results:**

Under a paradigm where two pure sounds 300 ms in duration and 800 or 840 Hz in frequency were presented in a specific order at an even probability, cortical responses to each sound were measured with magnetoencephalograms. Sounds were grouped to five events regardless of their frequency, 1D, 2D, and 3D (a sound preceded by one, two, or three different sounds), and 1S and 2S (a sound preceded by one or two same sounds). Whereas activation in the planum temporale did not differ among events, activation in the superior temporal gyrus (STG) was clearly greater for the different events (1D, 2D, 3D) than the same event (1S and 2S).

**Conclusions:**

One presentation of a sound is enough to shape a memory trace for comparison with a subsequent physically different sound and elicits change-related cortical responses in the STG. The STG works as a real-time sensory gate open to a new event.

## Background

The quick detection of a change in the sensory environment is very important to survival. Studies using functional magnetic resonance imaging (fMRI) [1] and magnetoencephalography (MEG) [2,3] have demonstrated change-specific cortical activation in the auditory, visual and somatosensory systems. Therefore, one can assume the presence of a system that facilitates detection of a change, orientation to the new event and a subsequent behavioral response. Since the detection of a sensory change is based on a comparison between the past and present sensory status, the change-detecting system should relate closely to short-lasting sensory memory. Sensory memory, and echoic memory for auditory sensory memory, in this paper means faculties of the human brain that can hold sensory information in a very accessible state temporarily [4-7].

In the auditory system, the mechanism of change-detection or its relation to echoic memory has been studied using mismatch negativity (MMN) [6-11] or Change-N1, a subcomponent of N1 [12-16]. Usually, MMN is investigated by comparing event-related potentials (ERPs) to a frequently presented standard stimulus and a rare deviant stimulus under an oddball paradigm, and is taken to reflect a preattentive memory-based comparison process [6]. Change-N1, which is elicited by a sudden change in a continuous tone and peaks approximately 100 ms after the onset of the change, has been also used to investigate higher auditory processes [12-16]. Although Change-N1 differs from MMN in that MMN does not contain the N1 component, Change-N1 is also considered to relate to auditory store [12-17]. Provided that sensory memory works to pick up sensory changes by updating the current sensory status in real-time, activation of the change-detecting system by any sensory change would be reflected in MMN or Change-N1. Indeed, the magnitude of change-related cortical activation reflects the strength of echoic memory of the past event to be compared [17]. However, according to the prevailing view, several presentations of a standard stimulus are needed to elicit MMN (for review, see [18]). To understand mechanisms of the change-detecting system, that is, to know how the brain recognizes a sensory change, this matter seems very important. It is also important to know whether change-related brain responses can be an objective index of sensory memory.

We hypothesized that sensory memory works in real time and therefore, the single presentation of a sound is enough to develop it. In the present study, we investigated whether the brief presentation of a sound forms sensory memory (echoic memory) that can be indexed by a change-related cortical response. The change-related cortical response in the auditory system 1) is elicited by any kind of auditory change including the onset and offset of a sound [19,20], 2) reflects the magnitude of the physical difference between present and past sounds, and 3) reflects the strength of echoic memory established for the previous sound [17]. In addition, the change-related response is little affected by the subject's attention, and is stably elicited during a long time course. Therefore, it seems useful to investigate echoic memory. To investigate whether a deviant sound following a single standard sound elicits a change-related response, we used a stimulation paradigm with two pure tones at an even probability. If echoic memory works in real time, a sound followed by a different sound would evoke a change-related cortical response. Although recently a multi-feature paradigm without a simple repetitive standard sound or even without a standard sound has been used to shorten the recording time for MMN [21-23], the present paradigm is different in that the probability of two sounds physically differing in a stimulus feature is even.

The present results clearly showed the real-time nature of echoic memory and the change-related cortical responses based on it. A model to explain the relationship between echoic memory and change-related cortical activation is discussed.

## Results

### Evoked waveforms

Two pure tones 300 ms in duration and 800 Hz or 840 Hz in frequency were presented in a specific order with an even probability. Under this paradigm, trials were grouped into two categories according to whether a sound was different from the previous sound, that is, same (SAME) and different (DIFF) trials. In the SAME trials, a sound was preceded by one (1S) or two (2S) sounds of the same frequency. In the DIFF trials, a sound was preceded by either of one (1D), two (2D), or three (3D) sounds of a different frequency (3D). Therefore, there were five events. The probability of the occurrence of the five events, 1D, 2D, 3D, 1S and 2S, was 1:1:1:2:1. In the present study, a comparison was made among the four equiprobable events.

**Figure 1:**
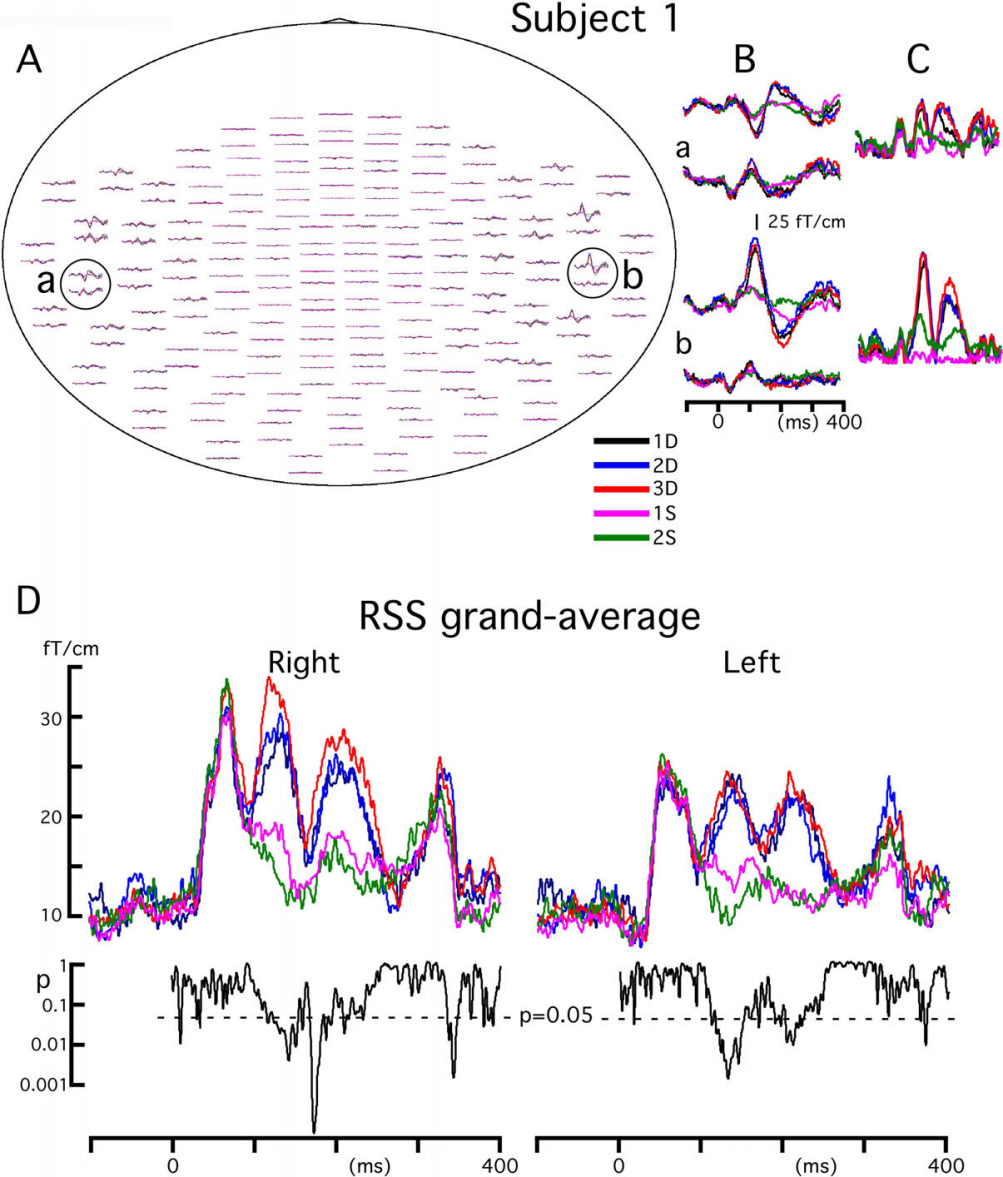
**Magnetic responses to the five events**. A ~ C, data from a representative subject. A), top view traces of all the sensors. B), enlarged waveforms of selected sensors indicated by circles in A, C), root sum square (RSS) waveforms obtained from selected pair gradiometers. D, grand-averaged RSS waveforms of the five events. The lower trace indicates the results (p value) of the one-way repeated measures ANOVA at all sampling points, which evaluates whether the RSS value differs significantly among events.

Figure 1A ~ C shows magnetic responses to the five events in a representative subject. Evoked fields were strikingly similar among the five events up to around 100 ms after the onset of stimulation, while the amplitude of the response clearly changed later on. That is, the main activity peaking at around 130 ms was larger for the DIFF trials than SAME trials. In each subject, the location in both hemispheres with the maximum root sum square (RSS) of the signals of pair gradiometers was selected. Procedures for the RSS analysis are shown in Figure 1: selection of the sensors' locations (1A), waveforms of pair gradiometers (1B), and RSS waveform (1C). Figure 1D shows grand-averages of the RSS waveforms across subjects. The RSS waveforms were almost the same up to 100 ms while clearly different at around 100 ~ 300 ms. Results of one-way repeated measures ANOVAs at each sampling point showed depression of the p value during this latency period indicating a significant difference in the RSS value among events. To evaluate hemispheric differences in amplitude, we compared RSS values of both hemispheres for each condition by using a two-tailed paired t-test. Results showed that the amplitude was significantly larger in the right hemisphere for 2D (192 ~ 197 ms) and 3D (183 ~ 202 ms).

**Figure 2:**
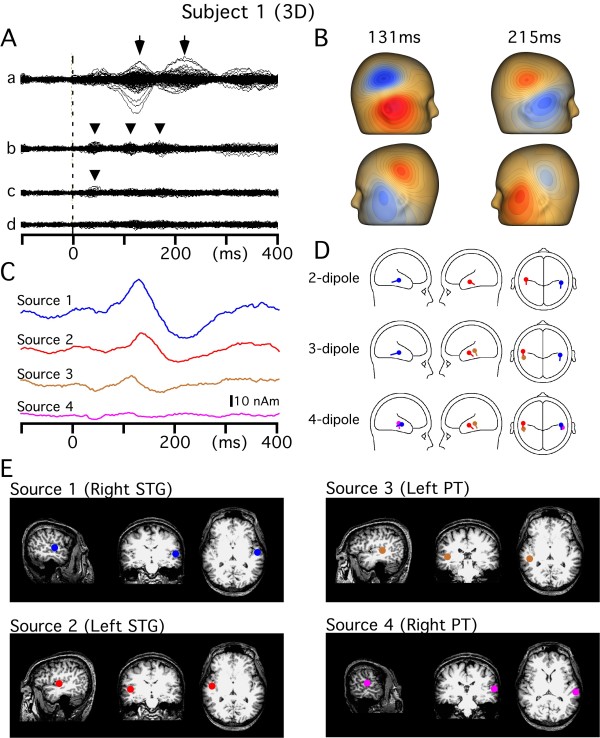
**Procedures for the multi-dipole analysis**. Data from a representative subject. A), superimposed waveforms recorded from 204 gradiometers. Aa shows original waveforms and Ab~d shows the residual waveforms not explained by a model. B) schematic drawings of the location and orientation of each dipole. C), source strength as a function of time of each dipole. D), the location and orientation of each dipole. E), the location of each dipole on the subject's own MR images. PT, planum temporale; STG, superior temporal gyrus.

Next, we performed a multi-dipole analysis to separate the cortical sources and know the time course of the activity of each. Figure 2 shows the procedures used for the analysis. Although the topography at all latency points of the original waveform (Aa) showed a dipolar field distribution consistent with two symmetric dipoles directed infero-posteriorly (B), two-dipole models left some clear component unexplained, suggesting more than two dipoles. Therefore, we started the analysis with two tentative dipoles (Source 1 and 2) that provided the best GOF value. In this case, a two-dipole model calculated at 131 ms (indicated by an arrow) showed the best GOF value (96.0%). Dipoles were estimated to be located in the antero-lateral part of Heschl's gyrus in both hemispheres. This location is more anterior and lateral to the primary auditory cortex and often appeared to lie in the gyrus of the brain surface rather than in the transverse gyrus (Figure 2E). Therefore, we labeled this source the superior temporal gyrus (STG). Figure 2Ab shows magnetic fields that were left unexplained by the two-dipole model. Then, a third dipole to best explain the residual waveform was searched for, and estimated to be located on the supratemporal plane slightly posterior to Heschl's gyrus, a region corresponding to the planum temporale [24]. This source was labeled PT. On addition of the third source (Source 3), the residual magnetic components at around 120 ms and 170 ms disappeared (Ac). This source significantly improved the fit, for example, increasing the GOF value from 85 to 93% (F = 2.1, p = 2.3 × 10^-6^) at 115 ms. Figure Ac shows residual waveforms that were left unexplained by the three-dipole model. After the location and orientation of Source 2 were slightly adjusted to provide the best GOF value with the existence of Source 3, the best fourth dipole to explain the residual waveform was searched for, and estimated to be located in an area slightly posterior to Source 1. Although the activity of this dipole was weak, the improvement in fit was significant (F = 1.7, p = 2 × 10^-4^). After fitting four dipoles, no clear residual component was seen in the residual waveform (Ad). Although some other sources were estimated according to our criteria in a few subjects, such as the anterior cingulate cortex and inferior frontal gyrus, the following analyses were performed on these four sources that were present in all the subjects.

**Figure 3:**
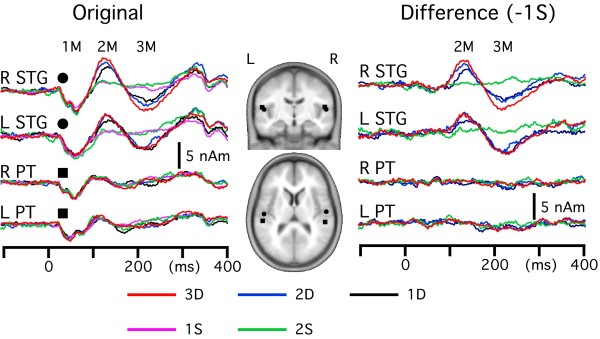
**Grand-averaged original and difference waveforms of each cortical activity in the five events**. The mean location of the source is shown in slices of a standard brain. The difference waveform was obtained by subtracting the 1S waveform from those of the other four events.

After the four cortical sources were obtained by the multi-dipole analyses for each subject, the peak latency and amplitude of each activity were compared among the four equiprobable events using ANOVAs. The grand-averages of a source's strength as a function of time are shown in Figure 3. As shown in Figure 3, both STG and PT activities consisted of three polarity-reversed components, 1M, 2M and 3M. At first, the peak latency and peak amplitude of each component were compared among four equiprobable events, 1D, 2D, 3D and 2S. As for the peak latency, results of one-way repeated measures ANOVA showed a significant difference among events only for 3M of the right STG activity (F = 3.5, p = 0.03). That is, the peak latency of 2S was shorter than that for the DIFF trials (Table 1). The peak amplitude of the PT activity did not differ among events for any component. On the other hand, the peak amplitude of 2M and 3M of the STG activities was significantly different (p = 0.03 ~ 0.0006) among events. That is, the amplitude was largest for 3D, followed by 2D, 1D and 2S.

**Table 1:** Peak latency and amplitude of each cortical activity

Peak Latency (ms)												
	STG						PT					
		
	Left			Right			Left			Right		
		
	1M	2M	3M	1M	2M	3M	1M	2M	3M	1M	2M	3M

1D	60	139	212	63	140	216	56	127	202	62	115	188

2D	62	137	216	66	133	223	57	126	198	62	116	184

3D	62	133	216	67	137	224	56	121	198	60	114	179

1S	58	134	211	64	137	204	59	124	193	62	114	173

2S	60	133	205	58	134	212	56	122	186	64	113	177

Peak amplitude (nAm)												

	STG						PT					
		
	Left			Right			Left			Right		
		
	1M	2M	3M	1M	2M	3M	1M	2M	3M	1M	2M	3M

1D	8.6	6.7	5.5	10.7	9	4.9	5.3	3	4.2	5.4	3.8	6.7

2D	7.9	8	5.3	7.5	10.5	5.6	5.7	4	3.9	6.3	4.1	7.3

3D	7.9	8.7	6.8	8.7	11.6	7.4	6.3	3.8	4.4	5.7	4.7	8.2

1S	9.4	3.4	1.6	7.7	5	1.3	5.6	2.9	1.9	7.1	2.6	4.9

2S	10.6	4.4	1.8	9.4	5.6	0.7	6.5	4.7	3.3	5.9	3.2	6.5

In standard MMN studies, MMN is seen as a negative displacement at the frontcentral electrodes in the difference wave obtained by subtracting the response to standard stimuli from that to deviant stimuli [9]. Therefore, next, difference waveforms were obtained by subtracting the source strength waveforms of 1S from those of 3D, 2D and 1D for comparison with previous studies using a subtraction method. The right panel in Figure 3 shows grand-averaged difference waveforms. As expected from the results above, the difference waveform of the STG source showed two components, 2M and 3M, but there was no clear activity for the PT source. There was no consistent activity in the difference waveform of 2S. When the peak latency and peak amplitude of 2M and 3M were compared among the three DIFF events, the latency was shorter and the amplitude was larger for 3D, 2D and 1D in this order (Table 2). Since the grand-averaged RSS waveform in Figure 1 and source strength waveform in Figure 3 showed an amplitude difference between hemispheres, we evaluated the difference using a paired t-test for each component (1M, 2M and 3M) for each cortical source (STG and PT). However, we could not find a significant difference for any case probably because of a large inter-individual difference.

**Table 2:** Peak latency and amplitude of the difference STG activity

	Peak latency (ms)				Peak amplitude (nAm)			
		
	Left		Right		Left		Right	
	2M	3M	2M	3M	2M	3M	2M	3M

1D	141	223	139	227	6.5	7	5.6	6.1

2D	137	220	136	221	7.3	7.3	7.7	6.5

3D	130	218	133	220	7	7.6	8.8	8.1

Results of discriminant analyses showed no significant differences in the source's location between any pair of events for any cortical source (p > 0.64). The mean coordinates of the source (average of all events) across subjects were as follows: Right -STG (52.7, -12.9, 10.5), Left-STG (-49.8, -16.8, 9.8), Right-PT (51.0, -30.0, 13.0), Left-PT (-52.7, -28.0, 12.7). The mean location of each source is shown on MR slices of a standard brain in Figure 3.

## Discussion

### Memory trace strength determines the amplitude of change-related responses

The present results clearly showed that the single presentation of a sound shaped a memory trace that could be used for comparison with a subsequent different sound as has already been proposed [25]. Under an oddball paradigm, so-called fresh-afferent neuronal activity might help to shape MMN [18]. That is, the repetition of a standard stimulus leads to adaptation of the cells contributing to the standard-elicited N1 (attenuated N1) but leaves the deviant-selective cells unadapted (enhanced N1). In the present study, this seems unlikely because two pure tones were presented at an even probability. The fact that the PT activity and early part of the STG activity did not differ in amplitude among events is also consistent with the idea that enhanced activation in the STG is not due to a different degree of adaptation of frequency-specific afferents. In an MEG study, Maess et al. [26] demonstrated that the late part of the change-related response evoked by a frequency deviance is based on a cognitive, comparator mechanism, which seems to correspond to the enhanced STG activity in the present study.

**Figure 4:**
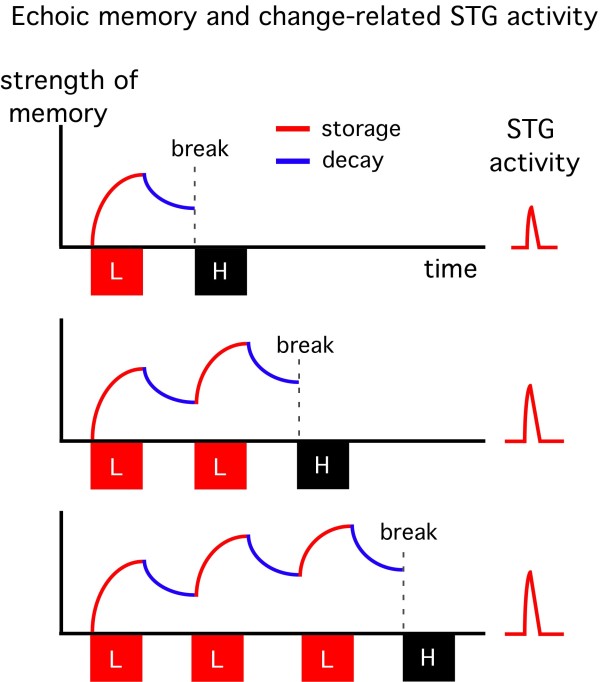
**A model to explain the relation between echoic memory and change-related cortical activity**. The vertical axis and horizontal axis indicate the strength of echoic memory and time, respectively. The model is based on the present and previous findings that a single presentation of a sound forms an echoic memory and the temporal representation of both the storage and decay of echoic memory is logarithmic [17]. In this model, echoic memory develops during presentation of the sound and decays during the silent interval in a logarithmic fashion, and the amplitude of the change-related STG response is linear to the strength of memory at the breaking point (onset of the deviant).

**Figure 5:**
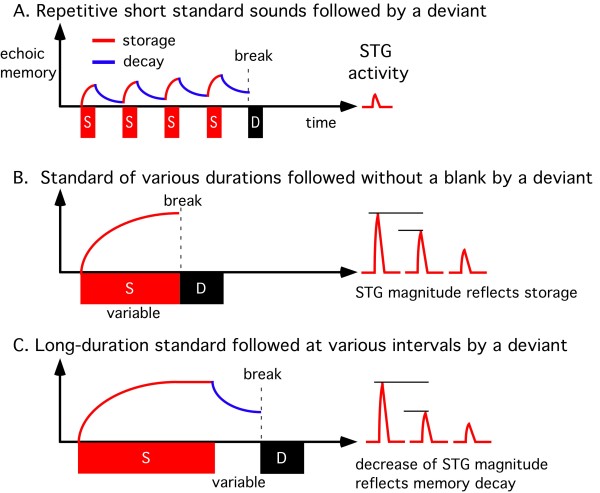
**Echoic memory and change-related cortical response under various paradigms**. A) repetitive standard sounds followed by a deviant, the so-called oddball paradigm usually used to obtain MMN. When the duration of the standard is short, the increase in memory strength during repetition of the same standard sound should be minimal, because of the small difference between storage and decay for each sound, which would result in a weak change response in spite of a long recording time. It should be noted that a deviant (rare) sound can be a standard for the next standard (frequent) sound. B) a standard sound followed without a blank by a deviant. Since memory decay does not occur, the amplitude of the change response reflects the degree of memory storage [17,20]. This paradigm would be useful when a patient possibly suffers from memory storage abnormalities. C) a long-duration standard followed by a deviant. When the duration of the interval between the standard and deviant is increased, the evoked change response decreases because of the decay [17]. This paradigm would be useful to detect a faster than normal decay of memory in a certain patient. It is important to note that there may be a group of patients in which the change-detecting system itself is disturbed. In such cases, any kind of stimulation paradigm can evoke only a weak, if any, change response. One such candidate is schizophrenia. In schizophrenics, it is known that both the On-response and MMN is weak, and in addition, sound-evoked responses are resistant to repetition (little activity to adapt).

Our recent study suggested that the temporal representation of echoic memory is logarithmic for both storage and decay [17]. Taken together with the present findings, we propose the model shown in Figure 4 to explain the relationship between echoic memory and the change-related STG activity. In the model, the strength of a memory trace at a given time is determined by memory storage and decay, and the magnitude of the change-related STG activity reflects the memory's strength at the time when a physically different sound breaks the trace. In this model, the slope of both memory storage and memory decay is not identical among the 1D, 2D and 3D events, since the cortical representation of echoic memory is not linear [17]. That is, the slope of storage becomes smaller at a stronger memory level while that of decay becomes sharper at a stronger memory level. This well explains the present finding that the amplitude of the STG activity of the 3D event was not three-fold that of 1D while the total duration of memory storage was three times that for 1D. Although the model may be insufficient (for example, it does not include the degree of difference between the two sounds), it well explains many reported data relating to change detection. Figure 5 shows models to explain the relationship between echoic memory and the change-related STG activity in other stimulation paradigms. Under a standard MMN paradigm, a deviant stimulus interspersed among a frequently presented standard stimulus evokes a MMN response. The idea that the amplitude of MMN at the breaking point is determined by summation of the storage and decay of echoic memory during repetition of a standard stimulus as drawn in Figure 5A is consistent with a previous finding that the amplitude of MMN increases with an increase in the number of standard stimuli [27]. If the standard sound is very short in duration, resultant change responses would be very small even when repeated many times because of the weak memory storage for each stimulus and the memory decay during the interval between stimulation. On the other hand, a deviant stimulus following a standard without a blank (5B) elicits a large change-related STG response [17,20] because of an absence of decay. Therefore, change-related responses reflect the memory storage function in this stimulation paradigm. The magnitude of the change-related response under such a paradigm increases with an increase in the duration of the standard with a logarithmic function [17,20]. When a deviant is presented after a long-duration standard with a blank, the amplitude of the change-related response decreases with an increase in the duration of the blank in a logarithmic fashion [17]. Therefore, change-related responses in Figure 5C reflect memory decay.

The model could lead to a new paradigm to test memory storage, memory decay and the change-detection system itself in clinical patients. We believe that there should be an appropriate method that is completed within a short time and provides data with a good s/n ratio or good test-retest reliability.

### STG as a sensory gate to pick-up a change

We consider that the STG activity in the DIFF trials is change-related or even change-specific. However, weak STG activity was also present in the SAME trials. Therefore, some discussion seems necessary to explain this finding. Any sensory change can be a new event and thus potentially drives the change-detecting system. In this regard, an onset of stimulation after a blank can be a new event against the silent background. If this STG activity (On-STG) depends on the memory trace of the presence of a previous sound, the On-STG activity in this study would be very weak because of the very short inter-trial interval (300 ms), but should be present. Recently, we demonstrated that the onset, offset and change in frequency of a sound all activate the STG and the amplitude of activity depends on the duration of the preceding condition with a logarithmic function [20], suggesting that any auditory change activates the STG through a memory-based comparison process. Both the onset and offset of a sound can be regarded as a large deviation of sound pressure from the preceding baseline. The view that the On-STG activity is a change-related response is well consistent with the stimulation rate-sensitive nature of the STG activity [28]. Although one may attribute the decrement of the On-response with stimulus repetition to habituation or adaptation instead of memory, using these terms to explain the phenomenon is meaningless if the definition of these terms is the response decrement itself. Habituation is one of the simplest forms of memory or learning (for review, see [29,30]). The decrease in On-STG activity with stimulus repetition, thus echoic memory, probably corresponds to short-term habituation that has been attributed to presynaptic inhibition in studies using *Aplysia *[30]. In mammals, there are also studies investigating the physiological significance of habituation. For example in the rat olfactory system, Kadohisa and Wilson [31] demonstrated that olfactory bulb cells continue to respond to a background odorant while anterior piriform cortex neurons rapidly and almost completely adapt to the background odorant but keep responding to a new odorant presented in addition to the background odorant. These findings indicate that the anterior piriform cortex acts as a filter driven by changing stimuli. Therefore, habituation allows neurons in the piriform cortex to respond selectively to a change in stimulus, contributing to separation of the target odorant from the background. Applying these findings in rats to the present study, STG neurons respond to each sound stimulus and 'adapt' rapidly (weak On-response), responding fully to a new sound (Change-response), therefore the STG works as a sensory gate to pick-up a change in sound among stable inputs from lower cortical areas. If adaptation means that the cortex stores past sensory information, adaptation can be regarded as one form of memory.

### Change-related responses in other sensory modalities

In a previous study using electroencephalograms [28], we showed that the main cortical areas sensitive to the interstimulus interval, that is, which easily adapt, are the STG for the auditory system, the opercular region for the tactile and pain systems, and the middle occipital gyrus (MOG) for the visual system. Given that a cortical area that adapts to stimulus repetition acts as a sensory gate open to a new event, one can expect these cortical areas to be responsible for the detection of change in each sensory modality. In fact, recent work from our lab demonstrated that the opercular region [32], MOG [3,33] and STG [19,20] are involved in change detection for the respective sensory modality. Activation in areas of the sensory cortex in response to a changing stimulus is consistent with the fact that in general, the sensory cortex is involved in the short-term storage of information [34]. We believe that future studies in animals will find neurons in corresponding areas that behave as a change detector like piriform cortex neurons in the paper by Kadohisa and Wilson [31].

### Hemisphere difference

Although grand-averaged waveforms showed that right hemisphere responses were larger in amplitude for both the RSS and source strength waveforms, results of statistical analyses showed no significant difference. One explanation might be the large inter-individual difference and small number of subjects in the present study. In a previous study on the mismatch response, right hemisphere dominance was demonstrated [35]. Since the present study used a fixed sound pressure level (70 dB SPL) for both ears of all the subjects, a subtle difference in the hearing threshold between ears might lead to differences in the right and left hemispheres. To evaluate the hemispheric difference precisely, careful adjustment of the sound pressure level for each ear of each subject appears necessary.

## Conclusions

One presentation of a sound is enough to shape a memory trace for comparison with a subsequent physically different sound and elicits change-related cortical responses in the STG. The STG works as a real-time sensory gate open to a new event.

## Methods

The experiment was performed on nine (one female and eight males) healthy right-handed volunteers, aged 27-46 years (33 ± 7). The study was approved in advance by the Ethics Committee of the National Institute for Physiological Sciences, Okazaki, Japan, and written consent was obtained from all the subjects.

### Stimulus and recordings

**Figure 6:**
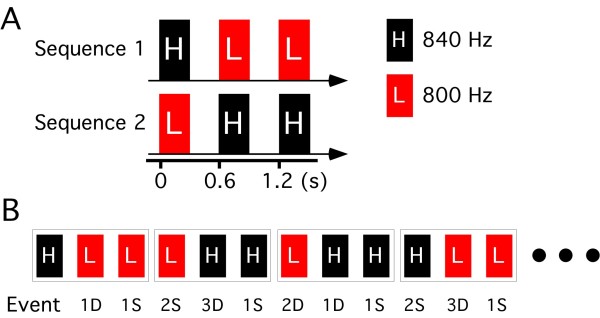
**Sound stimulus**. Stimuli were grouped into two categories according to whether the sound was the same (S) as or different (D) from the preceding stimulus. In the present paradigm, the probability of the S trial and D trial was even. According to the number of same or different preceding stimuli, S stimuli were grouped into two subgroups (1S and 2S), and D stimuli into three subgroups (1D, 2D and 3D).

Two pure tones 300 ms in duration (rise/fall, 5 ms) and 800 or 840 Hz in sound frequency were used. We made two sound sequences composed of three tones; 840 - 800 - 800 Hz (HLL) and 800 - 840 - 840 Hz (LHH) (Figure 6A). The interstimulus interval (ISI) between the tones (from offset to onset) was 300 ms. The two sequences, HLL and LHH, were randomly presented at an even probability with an interval of 300 ms during the experiment. Under this paradigm, the probability of each sound (800 or 840 Hz) was even and the probability of trials with a change (800 to 840 Hz or 840 to 800 Hz, DIFF trials) and trials without a change (SAME trials) was even. Among the DIFF trials, there appeared three types at an even probability; a trial with a tone preceded by a tone of a different frequency (1D), that preceded by two tones of a different frequency (2D)(LLH and HHL), and that preceded by three tones of a different frequency (3D)(LLLH and HHHL). Among the SAME trials, there were two types; a trial with a sound preceded by a tone of the same frequency (1S) and that preceded by two tones of the same frequency (2S)(LLL and HHH). Therefore, there were five types of events in this study, 1D, 2D, 3D, 1S and 2S with an occurrence probability of 1:1:1:2:1 (Figure 6B). None of the subjects could identify the sequence of sounds even when they listened carefully after the experiment.

The experiment was performed in a magnetically shielded room. Sound stimuli were presented through ear pieces (E-A-Rtone 3A, Aero Company, Indianapolis, IN) binaurally at 70 dB SPL. Throughout the experiment, subjects were instructed to watch a silent movie projected on a screen 1.5 m in front of them and to ignore the sound.

Magnetic signals were recorded using a 306-channel whole-head type MEG system (Vector-view, ELEKTA Neuromag, Helsinki, Finland), which comprised 102 identical triple sensor elements. Each sensor element consisted of two orthogonal planar gradiometers and one magnetometer coupled to a multi-superconducting quantum interference device (SQUID) and thus provided 3 independent measurements of the magnetic fields. In this study, we analyzed MEG signals recorded from 204 planar-type gradiometers. These planar gradiometers are powerful enough to detect the largest signal just over local cerebral sources. The signals were recorded with a bandpass of 1-200 Hz and digitized at 1001 Hz. The analysis was conducted from 100 ms before to 400 ms after the onset of each stimulus. The 100 ms pre-stimulus period was used as the baseline. Epochs with MEG signals larger than 2.7 pt/cm were rejected from the averaging. Epochs for four equiprobable events (1D, 2D, 3D, and 2S) were averaged at least 350 times, and therefore around 700 times for 1S.

### Analysis

First, we calculated vector sums from the longitudinal and latitudinal derivations of the response recorded on the planer-gradiometers at each of the 102 sensors' locations. This was obtained by calculating the root sum square (RSS) of MEG signals of two gradiometers at a sensor's location as described previously [36]. RSS waveforms were obtained for all 102 sensors' locations and we selected one location for each hemisphere with the maximal amplitude at a latency of 100 ~ 150 ms (major MEG component). After obtaining five RSS waveforms of each hemisphere for each subject, a one-way repeated measures ANOVA was performed among four equiprobable events, 1D, 2D, 3D and 2S, at each sampling point (about 400 points) to test whether recorded MEG signals were significantly different in amplitude at a certain latency among the four events.

Next, we performed a multi-dipole analysis using the brain electric source analysis (BESA) software package (NeuroScan, Mclean, VA) to separate several temporally overlapping cortical sources. Model adequacy was assessed by examining: 1) percent variance [37], 2) F-ratios (the ratio of reduced chi-square values before and after adding a new source) [38] and 3) residual waveforms (the difference between the recorded data and the model), as described elsewhere [39,40]. The integral probability of obtaining a F-ratio equal to or greater than the obtained value was calculated to evaluate whether a model with a larger number of dipoles represents a statistically significant improvement of fit over a model with a smaller number of dipoles. When a p value was smaller than 0.05, we considered the new dipole as significant. These calculations gave the three-dimensional (3D) location, orientation, and strength of the ECD in a spherical conductor model, which was based on each subject's magnetic resonance imaging (MRI, Siemens Allegra, 3.0-T) to show the source's location. Sources were superimposed on the individual MR images by using the head position indicator (HPI) system. The location was transformed into Talairach coordinates by BESA and Brain Voyager (QX 1.4, Maastricht, The Netherlands). BESA uses a spherical four-shell model (the brain, cerebrospinal fluid, bone and skin).

A one-way analysis of variance (ANOVA) was used for statistical comparisons of the latency and amplitude of each cortical activity among four equiprobable events, 1D, 2D, 3D, and 2S. The statistical significance of the source's location among events was assessed by a discriminant analysis using x, y, and z coordinates as variables. P values less than 0.05 were considered to be significant.

## Abbreviations

MEG: magnetoencephalogram; MMN: mismatch negativity; MOG: middle occipital gyrus; PT: planum temporale; RSS: root sum square; STG: superior temporal gyrus

## Authors' contributions

KI contributed to planning the study, data collection and analysis, and drafting the paper. TU, KY, NO, MN and EM contributed to data collection and analysis. YT contributed to constructing devices. TK and RK contributed to drafting the paper. All authors read and approved the final manuscript
